# Supplementary material for: Complementary environmental analysis and functional characterization of lower glycolysis-gluconeogenesis in the diatom plastid
Source: Plant Cell. 2024 Jun 6;36(9):3584–610. doi: 10.1093/plcell/koae168 (PMC11371179; doi:10.1093/plcell/koae168)
Supplement: koae168_Supplementary_Data [file tpc.00347.2024-s10.pdf › tpc.00347.2024-s10.pdf]

THE PLANT CELL

Complementary environmental analysis and functional characterization of a plastid diatom lower glycolytic-gluconeogenesis pathway

Richard Dorrell, Youjun Zhang, Yue Liang, Nolwenn Guéguen, Tomomi Nonoyama, Dany Croteau, Mathias Penot, Sandrine Adiba, Benjamin Bailleul, Valerie Gros, Juan Pierella Karlusich, Nathanaël Zweig, Alisdair Fernie, Juliette Jouhet, Eric Marechal, and Chris Bowler

Review Timeline:

Submission Date:	09-Jan-2024
Editorial Decision:	15-Feb-2024
Resubmission Received:	10-Apr-2024
Editorial Decision:	12-May-2024
Revision Received:	14-May-2024
Accepted:	14-May-2024

Dr. Richard G Dorrell
Institut de Biologie de l'Ecole Normale Supérieure (IBENS)
Paris
France

Dear Dr. Dorrell:

Thank you for choosing to send your manuscript entitled "Environmentally-informed functional characterization of a plastid diatom metabolic bridge of mitochondrial origin" for consideration at The Plant Cell. Your submission has been evaluated by members of the editorial board as well as expert reviewers in your field, and we regret to inform you that we are not able to recommend the publication of this manuscript in its current form. We have had input from multiple scientists and we have solicited post-review comments as well.

That said, during the post-review consultation session, we also agreed that if you could address the major points raised by the reviewers, we would encourage a resubmission. This may be treated as a new submission, but we would attempt to use at least some of the same reviewers. Nevertheless, reviewers will be asked to assess as a new manuscript (i.e. are the claims fully supported by the data and do the results presented move the field forward?), and not only whether previous reviewer comments have been addressed.

In general, it appears that there was genuine enthusiasm for the advances reported in the revised manuscript. However, there are still clarifications required in several places. There were concerns about the overreliance on the in vitro enzyme activity data, as well as a continued speculation on the adaptive value for this metabolic alteration in diatoms. Overall, the reviewers offered complementary, constructive criticisms that should help you and your coauthors to clarify the results, modify your discussion and focus the title better toward the more conclusive aspects of the manuscript while toning down the more speculative parts.

It will be important to convince the editors and reviewers that the study adds significant new understanding(s) of mechanisms or processes, or otherwise represents a significant advance in the field, and that the major claims made are fully justified by the data presented. This includes careful consideration and explanation of the various controls used in experiments, the extent and manner of replication, and the statistical analyses used. Sampling methods and the nature of "biological replicates" should be described precisely (i.e. different plants, parts of plants, pooled tissue, independent pools of tissue, sampled at different times, etc.), along with a clear description of and rationale for any statistical analyses conducted. The reader should know exactly what was sampled; what forms the basis of the calculation of any means and other statistical variables and parameters reported. This is also necessary to ensure that proper statistical analysis was conducted.

The Plant Cell now requires authors to complete and submit an author revisions checklist upon submission of a revised manuscript. The aim of the checklist is to aid authors in preparing a high-quality manuscript, facilitate the review and assessment of revised manuscripts, and help to ensure that journal standards are maintained across the board. If your manuscript is accepted, the completed checklist will be published as supplemental material attached to the article online. Please download a copy of the checklist (a fillable PDF form) at this link, for submission with your revised manuscript: https://tpc.msubmit.net/html/Author_Revisions_Checklist.pdf.

Note also that supplemental materials should be restricted to large datasets and tables, presentation of replicates, and validation of reagents, methods, or genotypes. Any data that are used to support the main claims must be in the main manuscript. Supplemental figure legends must indicate what figure in the main manuscript is supported by the supplemental data presented.

We thank you for your interest in and support of The Plant Cell. We look forward to your revised manuscript and hope that we will be able to issue a positive decision in the future.

Sincerely,

The Plant Cell Editorial Board

-- Reviewer comments:

Reviewer #3 (Comments for the Author):

I appreciate the efforts, the authors have taken to address the suggestions made by the reviewer. I will only comment on some

of my my original comments, not those of the other reviewers.
In my previous report I had raised several concerns regarding the manuscript, and some of them now have been addressed by the authors. One major issue was the lack of a clear proof that plastid glycolysis in diatoms indeed either is an adaptation or allows adaptation to high latitude conditions. The authors could not really solve this issue, mostly because the mutant lines meanwhile showed suppression of phenotypes, which is why for instance competition experiments that could demonstrate the advantage of plastidal glycolysis, are not feasible any more. Instead the authors modified and softened their claims in this direction by changing title and the text accordingly, so that the discussion of the outcome is justified.
I also appreciate that upon my comment, the authors added enzymatic analyses of cpENO and cpGAM expressesd in E.coli, and demonstrated that the enzymatic reaction may occur in both directions. This though is not too surprising, considering the nature of the enzymes and their reactions. Fig. 7A shows the respective activities of the combined enzymes in an enzymatic assay, however, in the text there is no description indicating how this compares e.g. to the respective cytosolic enzymes or to literature data. Furthermore, the reader cannot evaluate the data as they are given as relative reaction rates, not in the usual form as rate (like µmol substrate per time and amount of enzyme. The only mentioned outcome is that "the measured reaction rates were almost 1.5 times greater in the glycolytic than gluconeogenic direction". Here it also would be helpful to learn what this means with respect to the message of the manuscript when compared to literature data.
Finally, the new title though "Environmentally informed functional analysis..." sounds a bit odd to me, because "environmental data" and "functional analysis" in my view are two different aspects of investigation. I am not sure whether fusing the terms is helpful and will give a clear picture on the manuscript. I therefore would propose to change it to "Environmental data analysis and functional characterization of....."

Reviewer #4 (Comments for the Author):

The manuscript presented by Dorrell et al. reports on the role of two plastid enzymes (enolase and PGAM) involved in the glycolytic-gluconeogenesis pathway in the diatom Phaeodactylum tricornutum. They showed that this plastidial half pathway probably originated through the duplication of mitochondria-targeted isoforms and has a limited distribution across the algal tree of life. Using data from Tara Oceans campaigns, they showed that this pathway is mainly expressed at high latitudes and correlate this to photoperiod and temperature. Through the study of several mutants and their phenotype (growth, RNAseq, metabolites & lipids quantification) in 3 conditions (19{degree sign}C LD, 19{degree sign}C CL, 8{degree sign}C CL), the potential function of this pathway is highlighted by the authors. Even if the data presented do not assert that this pathway is a complete adaptation to high latitudes, I am convinced by the whole nice data set and concordance brought by the authors that this pathway is of importance and may contribute to the success of diatoms. The overall manuscript is very interesting, well written and has been really improved after the first revision. I have been through all the comments from the reviewers and found the modifications and answers from the authors quite convincing.
I have mainly two questions: (1) Why the plastid localization of PGAM1 Phatr3_J51404 was not performed? And (2) Fig S7: What is the difference between cpEnolase, surface (Fig S7i) and cpEnolase from Fig2? Same for cpPGAM?

There are also still many small mistakes (more specifically in the figures) that make the reading sometimes difficult! Also, there might have been some formatting problems for figures when the file was converted in pdf. Please make sure that figures are correct.
Line 75: suppress 'and' in front of 'chlorophyll'
Line 128: suppress 'in' in front of plastidial starch
Line 135: 'as opposed to the cytoplasm' for what or who? Reformulate as it is not clear.
Fig S1A and S1B: use the same color for PGAM only for a better visualization
Line 190 & 208: insert Supplemental Dataset instead of Dataset
Line 234: Phatr3_J50414 is not correct and should be corrected by 51404 I guess
Fig S3 and S4: add the key from Fig S2 for a better visualization
Fig S3: there is a problem in the color orange and brown in the Pelagophytes and Dictyochophytes proteins close to the plastidial Phatr3_J51404.
Fig S3: suppress the sentence 'S1, extending on the topology of Fig. 1C.' that is written to times.
FigS3: Phatr3_J 33819 is not correct in the figure and should be corrected by 33839.
In Fig 1B & 1C, Fig S2, Fig S3 and Fig S4: the thickness of the lines appears strangely sometimes and on Fig 1B, one of the nodes (the one going to mitoEnolase) is not well-centered (formatting problem?).
Fig 1A: cPGMA? please correct to cpPGAM
Fig 1B and 1C: titles of both figures (B-Enolase and C-PGAM) should be placed on the top of each figure not in the center of both - The color of Chrysophytes, Kelps in the key is different than the color in the tree which do not help the reader.
Line 249: Phatr3_J37201 is mentioned but why not Phatr3_J47096?
Line 265: ti et al. 2022 is not in the reference section
Fig S7: please correct ptEnolase & ptPGAM in cpEnolase and cpPGAM
Fig 2: Total transcriptome (left and not top) and genome (right and not bottom)
Line 341: suppress a coma and a bracket.
Fig 3B: problem with the y axis and statistical bars and stars!

Fig S9: correct "emcpy vector" in the caption. The title of the y-axis is not understandable
Fig S12: not cited in the manuscript and with formatting problem (stars not aligned...), maybe a problem of pdf conversion
Line 598: Fig S5 is not the right figure to be cited. It should be I guess Fig S2 or S3.

Reviewer #5 (Comments for the Author):

Diatom metabolism accounts for a significant amount of carbon assimilation, principally in cooler regions in the ocean, they are indeed primary producers and the topic of photosynthetic metabolism here is of high importance. There are several significant claims in the manuscript: i) the triose conversion steps for PEP production are present and active in the chloroplast of diatoms, and ii) their activity is linked to their environment. If sufficiently substantiated, these claims would be of immense value to the scientific community, given that triose utilization is possibly the most central function of metabolism of all living systems. For reasons echoed below, the authors would do well to acknowledge throughout that the role of the enzymes is somewhat speculative (unless understanding of Figure 2 can be improved - see below) and emphasize that here a number of pieces of evidence are provided to indicate the pathway in the chloroplast is complete in diatoms.

Since the original submission the authors have attempted to address skepticism from reviewers that can be summarized as: i) conclusion were based on too much correlative analysis, and ii) assertions that the diatom triose activity were focused to polar regions was not substantiated. Suggestions about metabolite levels being prone to artifacts because of the measurement approach and requiring kinetic and labeling studies were partially but incompletely addressed in the revision as 13C experiments with glycerol were unsuccessful. Further, pools would be distributed across organelles and quite challenging if not impossible to quantify within a specific subcellular location. It is noteworthy that two of the authors from Gohm are located where this expertise can be found.

Overexpression of the sequences in e coli would indicate function and could ensure that the enzymes were capable of operating with the correct direction (or both directions) for given substrates to product; however, the flux through any of these steps would not be known from such an activity analysis. Additionally, the flow through these steps is driven by concentration gradients (Hoppe et al., 1993; Stitt and ap Rees, 1979); thus, measurements of overexpression in e coli, and not isotope labeling of the diatom tissues would confirm a potential phosphoglyceromutase and enolase function but is unlikely to indicate anything further. I think the text on directionality (end of results) is appropriate and does not overstate the in vitro finding. As the authors point out, labeling would not inherently indicate the location of the production of PEP which is a focal point here, therefore even labeling analysis in diatoms may not be definitive unless the subcellular aspects could be resolved.

Are the claims fully supported? Do they move the field forward? The combination of phylogenetic analysis, subcellular localization prediction, and GFP localization provide sufficient evidence for me that the genes of interest are chloroplast-based.

The transcript levels of the genes in the field inferred from the Tara project and in the lab from knockout lines, are suggestive that they are actively expressed in conditions relevant to diatoms, though I was confused that changes to low temperature did not have an effect? Further, the lab diatom, presumably one that was molecular biology friendly, is not native to polar regions. Thus, the claims related to this role in the wild are weakened accordingly and statements around figure 2 should be extremely cautious (see Fig 2 comment further down for clarifications). For reasons the authors indicate in rebuttal, performing competition assays would be challenging and in the discussion at 630 they indicate this remains to be determined. If Figure 2 cannot be better explained so that it shows diatoms that don't express these genes at other parts of the globe, then the characterization that these genes are important for sub-polar should be emphasized to be speculative.

The included activity assessments establish function through production and purification from e coli, with the possible caveat that enzyme activities measured by spectrophotometer readouts of NADH could be insufficiently specific to the metabolism of interest. Nonetheless they are a supporting piece of evidence now provided.

Figure 2: Its possible I am not following your relative comparison, but are there no significant amount of diatoms near the equator? (Suppl Fig S1 also) If there are why not show the distribution of all diatoms and their expression within this picture, otherwise there is no reason to know if there are other diatoms at other places and whether they use these genes in their habitats? Its not compelling unless the diatoms expressing these genes are plotted relative to others not expressing these genes in warm environments. You indicate they are relative, but I don't see all diatoms are being plotted to show others for example near the equator that are not expressing the genes? Fig S7 is not helping my understanding of this. What you want to show is that the expression pattern in diatoms across the globe and support that expression is mostly in the sub-polar regions, but I do not understand what size fraction means and therefore I cannot interpret even the yellow vs orange and size of the bubbles in pictures like S7iii that seem to show some level of enolase near the equator and non-polar regions. What do size fractions in um have to do with expression of a gene?

Figure 3: not particularly compelling that light or temperature matters? Further at 8C, there is no difference - a cold condition... yet the KO line is a transformable line...and not a sub-polar line. I think at MOST you can infer from figure 2 a role in sub-polar conditions in the discussion and word carefully in results, but I remain skeptical of that role similar to prior reviews.

Figure 7B: consider just combining with 7A as bar plot activities - you cannot infer flux, as the triose phosphate interconversion is driven by concentration gradients which are unknown and would vary with condition. Plot the activities. Further, I think the point is that they have activity consistent with putative gene function of PGAM/ENO, not really that you can assess their reversibility in a biological context specific to a diatom in a particular environment, through an activity assay.

Its unfortunate you were unable to measure the substrates or products of the genes for the knockout relative to the WT, this could have been helpful, if they were being significantly used, and further could have supported your assessments for the role of these genes in cool conditions. Of course, metabolite levels may not be indicative of metabolism generally; but in a KO the immediate substrate and product are often affected. Fortunately, at least lower levels of pyruvate were indicated in the KO.

Minor:
A. I would alter the title to be something about the triose conversions of glycolysis to pyruvate in chloroplasts of diatoms?
B. Abstract 49: should state specifically 'for diatoms', we don't know what other species might have green chloroplasts with ENO/PGAM and whether they inherited from mitochondria, right?
C. Understanding of gluconeogenesis is not correct in line 72, gluconeogenesis is not for the production of starch rather sugars, consider phrasing carefully.
D. Figure 1D: yellow is green and red, but would be better to see each color and then merge at least for the example shown, rather than put in S5? I think this would make it easier for the reader.
E. Figure 3: specify the control is empty vector and not just WT.

Dear editors,

We thank you and the reviewers for the time and careful comments applied to our manuscript. As per our previous resubmission, we provide a detailed response to the supplied comments below in italic text with a double caret (“>>”) indent. We have confirmed that all of the new data associated with this revision are appropriately documented, and resubmit our previous copy of the Author Revision Checklist without modification from the primary resubmission.

In principle our revisions in this version of the text relate mainly to phrasing of the text and the arguments presented. Concerning the phrasing of the manuscript as a whole, we have chosen the revised title “Complementary environmental data analysis and functional characterization of a plastid diatom lower glycolytic-gluconeogenesis pathway”, reflecting that the *Tara* and *Phaeodactylum* analyses use different source data, but present similar conclusions regarding the roles of plastid glycolysis.

Elsewhere, we have, added new computational data, alongside some previously performed analysis that had been removed from an earlier iteration of the paper, and detail these findings in a new. Fig. 2, S6, S7, S10 and S12. Critically, these include evidence from published transcriptomes and qRT-PCR that cpEnolase and cpPGAM1A are transcriptionally induced by long days and low temperatures in *Phaeodactylum* despite its mesophilic origin. We feel this is sufficient to bridge some of the questions proposed between the use of *Phaeodactylum* as a model system and the consideration of global environmental expression trends from *Tara* Oceans data. We additionally show from published mass spectrometry data that the mutated genes cpEnolase and cpPGAM1A are likely the predominant isoforms in the *Phaeodactylum* chloroplast.

We further show using meta-genome assembled genomes from the *Tara* Oceans dataset that plastidial lower half glycolysis is predominantly associated with diatoms endogenous to high latitude environments. We further show an independent recruitment of lower half glycolysis to the chloroplasts of the distantly related green alga *Micromonas*, where it is also uniquely associated with high latitude ecotypes. We feel that these data provide some preliminary evidence in support of the fitness consequences of this pathway in high latitude habitats, but have nonetheless chosen to tone down the conclusions drawn with regard to adaptation due to the probable ancestral origin of this pathway within the diatom lineage.

We hope that the new computational data, and moderated phrasing of our revised manuscript text, are sufficient for publication, and look forward to hearing from you in due course.

With best wishes,

Richard G. Dorrell
Laboratory of Computational and Quantitative Biology, Sorbonne Université, Paris

Dear Dr. Dorrell:

Thank you for choosing to send your manuscript entitled "Environmentally-informed functional characterization of a plastid diatom metabolic bridge of mitochondrial origin" for consideration at The Plant Cell. Your submission has been evaluated by members of the editorial board as well as expert reviewers in your field, and we regret to inform you that we are not able to recommend the publication of this manuscript in its current form. We have had input from multiple scientists and we have solicited post-review comments as well.

That said, during the post-review consultation session, we also agreed that if you could address the major points raised by the reviewers, we would encourage a resubmission. This may be treated as a new submission, but we would attempt to use at least some of the same reviewers. Nevertheless, reviewers will be asked to assess as a new manuscript (i.e. are the claims fully supported by the data and do the results presented move the field forward?), and not only whether previous reviewer comments have been addressed.

In general, it appears that there was genuine enthusiasm for the advances reported in the revised manuscript. However, there are still clarifications required in several places. There were concerns about the overreliance on the in vitro enzyme activity data, as well as a continued speculation on the adaptive value for this metabolic alteration in diatoms. Overall, the reviewers offered complementary, constructive criticisms that should help you and your coauthors to clarify the results, modify your discussion and focus the title better toward the more conclusive aspects of the manuscript while toning down the more speculative parts.

It will be important to convince the editors and reviewers that the study adds significant new understanding(s) of mechanisms or processes, or otherwise represents a significant advance in the field, and that the major claims made are fully justified by the data presented. This includes careful consideration and explanation of the various controls used in experiments, the extent and manner of replication, and the statistical analyses used. Sampling methods and the nature of "biological replicates" should be described precisely (i.e. different plants, parts of plants, pooled tissue, independent pools of tissue, sampled at different times, etc.), along with a clear description of and rationale for any statistical analyses conducted. The reader should know exactly what was sampled; what forms the basis of the calculation of any means and other statistical variables and parameters reported. This is also necessary to ensure that proper statistical analysis was conducted.

The Plant Cell now requires authors to complete and submit an author revisions checklist upon submission of a revised manuscript. The aim of the checklist is to aid authors in preparing a high-quality manuscript, facilitate the review and assessment of revised manuscripts, and help to ensure that journal standards are maintained across the board. If your manuscript is accepted, the completed checklist will be published as supplemental material attached to the article online. Please download a copy of the checklist (a fillable PDF form) at this link, for submission with your revised manuscript: https://tpc.msubmit.net/html/Author_Revisions_Checklist.pdf.

Note also that supplemental materials should be restricted to large datasets and tables, presentation of replicates, and validation of reagents, methods, or genotypes. Any data that are used to support the main claims must be in the main manuscript. Supplemental figure legends must indicate what figure in the main manuscript is supported by the supplemental data presented.

We thank you for your interest in and support of The Plant Cell. We look forward to your revised manuscript and hope that we will be able to issue a positive decision in the future.

Sincerely,

The Plant Cell Board of Reviewing Editors

-- Reviewer comments:

Reviewer #3 (Comments for the Author):

I appreciate the efforts, the authors have taken to address the suggestions made by the reviewer. I will only comment on some of my my original comments, not those of the other reviewers.
In my previous report I had raised several concerns regarding the manuscript, and some of them now have been addressed by the authors. One major issue was the lack of a clear proof that plastid glycolysis in diatoms indeed either is an adaptation or allows adaptation to high latitude conditions. The authors could not really solve this issue, mostly because the mutant lines meanwhile showed suppression of phenotypes, which is why for instance competition experiments that could demonstrate the advantage of plastidal glycolysis, are not feasible any more. Instead the authors modified and softened their claims in this direction by changing title and the text accordingly, so that the discussion of the outcome is justified.

>>We thank the reviewer for their comments. To support our previous evidence that lower half plastidial glycolysis has important physiological roles in low temperature and long day conditions, we have added an additional analysis of Phaeodactylum gene expression shown in Fig. 2 and Fig. S7, as described below.

We had previously removed much of the discussion of adaptation from the manuscript text during our initial resubmission, and we have now removed this word from the Abstract as well. We have now also modified the Abstract to state "Considering experimental data, we show that this pathway has different roles dependent in particular on day length and environmental temperature, and show that it is expressed at elevated levels in high latitude oceans where diatoms are abundant.", thus avoiding any associative inference between meta-genomic data from polar diatoms and experimental data from Phaeodactylum, a mesophilic species. Finally, we have modified the subheadings of the Results sections to describe purely the analyses performed in each section, as opposed to our inferences of what these results suggest.

We are actually very hesitant to refer to plastid glycolysis as an adaptive feature in diatoms given its clear presence in the diatom common ancestor: to our understanding, it cannot be viewed as adaptive as it has not been gained by specific diatom species in response to specific environmental selection, and any differential presence of plastid glycolysis across the diatom tree would rather be driven by secondary loss from some species. We have tried to clarify this point in the Discussion, lines 876-877: "We are hesitant to state that plastid glycolysis is an adaptive feature of diatoms towards high latitudes, given that it apparently originated in a common ancestor of diatoms and several other algal groups ..this may be best identified through competition assays, e.g., between diatom species with different plastid carbon metabolism arrangements, or between Phaeodactylum knockout and control lines under each condition ". Reflecting that diatom plastid glycolysis may have different physiologically important functions in different regions of the global ocean, we have further stated in the Discussion that, "lower glycolysis-gluconeogenesis may have multiple functions in the diatom plastid, with different functions dependent on both light and temperature conditions" (lines 904-905).

I also appreciate that upon my comment, the authors added enzymatic analyses of cpENO and cpGAM expressesd in E.coli, and demonstrated that the enzymatic reaction may occur in both directions. This though is not too surprising, considering the nature of the enzymes and their reactions. Fig. 7A shows the respective activities of the combined enzymes in an enzymatic assay, however, in the text there is no description indicating how this compares e.g. to the respective cytosolic enzymes or to literature data. Furthermore, the reader cannot evaluate the data as they are given as relative reaction rates, not in the usual form as rate (like µmol substrate per time and amount of enzyme. The only mentioned outcome is that "the measured reaction rates were almost 1.5 times greater in the glycolytic than gluconeogenic direction". Here it also would be helpful to learn what this means with respect to the message of the manuscript when compared to literature data.

>>We thank the reviewer for their comments. We have converted the previously measured relative reaction rates into absolute values (moles per enzyme grams per second), using similar calculations as performed in (Zhang et al., Nature Communications 2020 doi: 10.1038/s41467-020-18234-w). We have provided these data in a modified Fig. 8, Fig. S20 and Table S6.

Concerning measured reaction rates of Enolase and PGAM in the literature, both are widely cited to have bidirectional reaction kinetics (as neither consume nor produce ATP), but we find few examples of direct measurements of the activity of either enzyme in each direction within the same study. The closest we can find to this are measurements from Krebs 1963 and Scrutton and Utter 1968, which measure rat kidney Enolase activity in glycolytic and gluconeogenic directions as a function of wet and dry mass, respectively. We have converted these rates into dry mass (using measured rat kidney relative water contents from Reinoso et al. 1997) and present these in lines 803-804: "measured rate of enolase in rat kidney tissue equivalent to approximately 14,000 µmol g dry weight-1 hr-1 in the glycolytic direction, and 20,000 µmol g dry weight-1 hr-1 in the gluconeogenic direction". We cannot find equivalent measurements for PGAM as (per the Scrutton and Utter study above) the measured reaction rates of this enzyme are exponentially greater than Enolase. We have also included meta-data of Km values for purified copies of these enzymes, stating in lines 759 to 762 "Equally, purified enolase and PGAM typically show greater affinity for 3-PGA than PEP, with a 5-8 fold difference in Km measured in mammalian, yeast and Trypanosoma brucei enzymes (Rider and Taylor, 1974; Hannaert et al., 2003)".

The innate activity of both Enolase and PGAM, given their probable reversibility, is more likely to be defined by substrate availability in each direction (see below) and so we have modified the results text to remove the emphasis on a greater innate glycolytic capacity of cpEnolase, as follows: "Previous studies project completely reversible (bidirectional) reaction kinetics with limited differences in the rates observed in the glycolytic versus gluconeogenic directions ... The measured reaction rates were effectively reversible, albeit marginally greater in the glycolytic than gluconeogenic direction" (lines 818-819).

Finally, the new title though "Environmentally informed functional analysis..." sounds a bit odd to me, because "environmental data" and "functional analysis" in my view are two different aspects of investigation. I am not sure whether fusing the terms is helpful and will give a clear picture on the manuscript. I therefore would propose to change it to "Environmental data analysis and functional characterization of....."

>>We agree with the reviewer, although have proposed the title "Complementary environmental data analysis and functional characterization of a plastid diatom lower glycolytic-gluconeogenesis pathway", as both components of our study independently point to plastid glycolysis phenotypes relating to light and temperature.

Reviewer #4 (Comments for the Author):

The manuscript presented by Dorrell et al. reports on the role of two plastid enzymes (enolase and PGAM) involved in the glycolytic-gluconeogenesis pathway in the diatom Phaeodactylum tricornutum. They showed that this plastidial half pathway probably originated through the duplication of mitochondria-targeted isoforms and has a limited distribution across the algal tree of life. Using data from Tara Oceans campaigns, they showed that this pathway is mainly expressed at high latitudes and correlate this to photoperiod and temperature. Through the study of several mutants and their phenotype (growth, RNAseq, metabolites & lipids quantification) in 3 conditions (19{degree sign}C LD, 19{degree sign}C CL, 8{degree sign}C CL), the potential function of this pathway is highlighted by the authors. Even if the data presented do not assert that this pathway is a complete adaptation to high latitudes, I am convinced by the whole nice data set and concordance brought by the authors that this pathway is of importance and may contribute to the success of diatoms. The overall manuscript is very interesting, well written and has been really improved after the first revision. I have been through all the comments from the reviewers and found the modifications and answers from the authors quite convincing.

>>We thank the reviewer for their kind comments. We have supported our initial Tara analyses with some further analysis of metagenome-assembled genomes, described in a new Fig. S12 and below, which suggest a concordant enrichment in algae possessing lower plastid glycolysis in high latitude regions. *We describe the implications and limitations of these associations in Discussion lines 869-870: "We thus tentatively propose that lower plastid glycolysis correlates specifically to diatom occurrence in high latitude* Tara *Oceans stations, with diatoms that lack identifiable copies of these proteins absent from these stations ... We are hesitant to state that plastid glycolysis is an adaptive feature (given that it) is retained in species such as* Phaeodactylum, *which is typically associated with intermediate latitudes"*

I have mainly two questions: (1) Why the plastid localization of PGAM1 Phatr3_J51404 was not performed?

>>We agree with the reviewer that information about this localisation should have been integrated into our study. In fact, cpPGAM1B has been experimentally localised to the plastid in Río Bártulos et al., 2018: we now cite this in lines 270-273: We note that both cpEnolase and cpPGAM1B have been independently localised with GFP to the P. tricornutum *plastid in a separate study (Río Bártulos et al., 2018).*

To assess whether cpPGAM1B and cpPGAM2 may have additional supplementary roles in the Phaeodactylum *plastid, we have added a new Fig. S6 that demonstrates that neither of these two enzymes are detectable in experimental proteomic data from the* Phaeodactylum *plastid, following Huang et al. 2023; and that their gene expression is not coordinated with those of cpEnolase or cpPGAM1A from meta-analyses of published gene expression data, according to Liu et al. 2022. We therefore clarify in the Results*

text (lines 302-303) that: "The coexpression of cpEnolase *and* cpPGAM1A *and accumulation of both encoded proteins in the plastid suggest that they possess linked metabolic functions"*

And (2) Fig S7: What is the difference between cpEnolase, surface (Fig S7i) and cpEnolase from Fig2? Same for cpPGAM?

>>We apologise for the technical oversight in how these data are presented: Fig. 2 (now Fig. 3) presents data from all depths collected during the Tara *Oceans expedition, and filtered only to remove particles < 0.8 µm or > 2000 µm. This gives a digestible overview of what organisms are present in each station and is typically used to synthesise global scale data in* Tara *Oceans, but may be insufficient to detect and quantify either very small organisms (due to lower DNA content than large cells) or very large ones (due to lower effective population sizes than smaller organisms).*

In parallel, therefore, we and other Tara *Oceans studies typically present abundances calculated for individual size fractions obtained by sub-filtration of collected water samples (previous Fig. S7; now Fig. S9). These allow greater visibility of individual organisms abundant in specific size ranges, and can help complement and provide context to the complete dataset. We have clarified these points in the Results text lines 380-384: "We observed these trends concordantly in both surface and deep chlorophyll maximum (DCM) samples from 0.8 – 2000 µm filtered … and in individual size fractions … suggesting broad reproductibiilty across diatoms independent of cell size and depth" and in the legends for Figs. 3 "These data provide a global overview of* Tara *Oceans meta-gene abundances, in complement to data from individual size fractions shown in Fig. S9." and Fig. S9: "These data, specific to individual diatom size classes, support global trends observed across all (unfiltered) size fractions and surface layers shown in Fig. 3."*

There are also still many small mistakes (more specifically in the figures) that make the reading sometimes difficult! Also, there might have been some formatting problems for figures when the file was converted in pdf. Please make sure that figures are correct.

>>We thank the reviewer for their careful scrutiny of our figures, and have corrected them manually for legibility. We will be happy to receive specific comments for revision of any figures in our manuscript if some aspects have been overlooked.

Line 75: suppress 'and' in front of 'chlorophyll'

>>Modified

Line 128: suppress 'in' in front of plastidial starch

>>Modified

Line 135: 'as opposed to the cytoplasm' for what or who? Reformulate as it is not clear.

>>Modified: "Diatoms are instead inferred…"

Fig S1A and S1B: use the same color for PGAM only for a better visualization

>>Modified. We have also placed the colour key at the top of the figure to cover both panels.

Line 190 & 208: insert Supplemental Dataset instead of Dataset

>>Modified

Line 234: Phatr3_J50414 is not correct and should be corrected by 51404 I guess

>>Modified

Fig S3 and S4: add the key from Fig S2 for a better visualization

>>Modified

Fig S3: there is a problem in the color orange and brown in the Pelagophytes and Dictyochophytes proteins close to the plastidial Phatr3_J51404.

>>Modified

Fig S3: suppress the sentence 'S1, extending on the topology of Fig. 1C.' that is written to times.

>>Modified

FigS3: Phatr3_J 33819 is not correct in the figure and should be corrected by 33839.

>>Modified

In Fig 1B & 1C, Fig S2, Fig S3 and Fig S4: the thickness of the lines appears strangely sometimes and on Fig 1B, one of the nodes (the one going to mitoEnolase) is not well-centered (formatting problem?).

>>We have moved the node as requested. The line thickness is defined by the program used to visualise the tree (FigTree) which works directly from the output nexus file. Line thickness relates to consensus support across the different substitution matrices employed, with thick lines indicating repeated recovery of the same clade: we have now stated this in the Figure 1 legend. We prefer not to modify the tree output files at this point as the scales on which they are shown were selected to allow optimal visualisation on one page, and each tree output has been manually reannotated in its current svg or pptx display.

Fig 1A: cPGMA? please correct to cpPGAM

>>Modified

Fig 1B and 1C: titles of both figures (B-Enolase and C-PGAM) should be placed on the top of each figure not in the center of both - The color of Chrysophytes, Kelps in the key is different than the color in the tree which do not help the reader.

>>Modified. *We have also moved the previous Fig. 1D to a new Fig. 2A to improve the layout of this figure.*

Line 249: Phatr3_J37201 is mentioned but why not Phatr3_J47096?

>>Modified. We focused on 37201 (e.g., for GFP localisation) as it branches within the diatoms, whereas 47096 seems more divergent and a sister to all diatoms, which we have also noted in the Results text.

Line 265: ti et al. 2022 is not in the reference section

>>We apologise- this was a typo and should have read Liu, Storti et al. 2022, which is cited. We have corrected this.

Fig S7: please correct ptEnolase & ptPGAM in cpEnolase and cpPGAM

>>Modified.

Fig 2: Total transcriptome (left and not top) and genome (right and not bottom)

>>We have changed the overall layout of Fig. 2 (now Fig. 3), such that metaG data are now shown in a lower panel B for both surface and DCM depths.

Line 341: suppress a coma and a bracket.

>>We have removed the comma prior to the reference, and rephrased the sentence to remove the need to put a reference (double bracket) in parentheses.

Fig 3B: problem with the y axis and statistical bars and stars!

>>We have corrected the figure.

Fig S9: correct "emcpy vector" in the caption. The title of the y-axis is not understandable

>>We have corrected the figure (now S13) legend and y-axis.

Fig S12: not cited in the manuscript and with formatting problem (stars not aligned...), maybe a problem of pdf conversion

>>We added a figure citation (now S16) in the Results text. The asterisks are integrated into the excel output (they are adapted bar labels) so should be correctly vertically arranged. We have nonetheless placed the horizontal axis intercept at 0.01 in each graph to improve its legibility, and reformatted the produced pdf directly in Inkscape.

Line 598: Fig S5 is not the right figure to be cited. It should be I guess Fig S2 or S3.

>>Reflecting our subsequent identification of lower plastid glycolysis in Micromonas MAGs, we have modified the text as follows: "with no detectable examples in primary red and few occurrences in green algae (Fig. S1A, S12)"

Reviewer #5 (Comments for the Author):

Diatom metabolism accounts for a significant amount of carbon assimilation, principally in cooler regions in the ocean, they are indeed primary producers and the topic of photosynthetic metabolism here is of high importance. There are several significant claims in the manuscript: i) the triose conversion steps for PEP production are present and active in the chloroplast of diatoms, and ii) their activity is linked to their environment. If sufficiently substantiated, these claims would be of immense value to the scientific community, given that triose utilization is possibly the most central function of metabolism of all living systems. For reasons echoed below, the authors would do well to acknowledge throughout that the role of the enzymes is somewhat speculative (unless understanding of Figure 2 can be improved - see below) and emphasize that here a number of pieces of evidence are provided to indicate the pathway in the chloroplast is complete in diatoms.

>>We thank the reviewer for their comments. We have, as discussed below, provided additional data (in Fig. 2B, S7, S10, and S12) to demonstrate evidence for flux through lower chloroplast glycolysis in diatoms considering both cellular and environmental data. We have globally modified the Discussion to emphasise which points are speculative or inferential, albeit supported by multiple lines of evidence in our data, e.g.,:

- *Lines 816-817: "It is possible that the occurrence of organelle-targeted isoforms of these enzymes is underestimated, e.g., due to lower sensitivity of diatom and plant-trained targeting predictors in other algal groups"*
- *Lines 843-844: "We are hesitant to state that plastid glycolysis is an adaptive feature of diatoms towards high latitudes"*
- *Lines 870-86671 "We thus tentatively propose that lower glycolysis-gluconeogenesis may have multiple functions in the diatom plastid, with different functions dependent on both light and temperature conditions"*
- *Line 875-876: "we suggest potential functions contributed by the lower plastid glycolysis-gluconeogenesis"*

Since the original submission the authors have attempted to address skepticism from reviewers that can be summarized as: i) conclusion were based on too much correlative analysis, and ii) assertions that the diatom triose activity were focused to polar regions was not substantiated. Suggestions about metabolite levels being prone to artifacts because of the measurement approach and requiring kinetic and labeling studies were partially but incompletely addressed in the revision as 13C experiments with glycerol were unsuccessful. Further, pools would be distributed across organelles and quite challenging if not impossible to quantify within a specific subcellular location. It is noteworthy that two of the authors from Gohm are located where this expertise can be found.

>>We have indeed now passed a further 55 13C-labelled diatom cell pellets generated for control versus glycolysis knockout lines under 19C CL conditions using machines available in Golm, but regret that we cannot detect sufficient 13C labels over the incubation timeframes requested by the original reviewers (<1 day) to be able to infer differences of flux in control versus knockout lines.

We have instead made a complementary suggestion in the Discussion (lines 986-989), which is to use inducible multiple knockouts targeting both lower chloroplast glycolysis and other metabolic pathways associated with the plastid to impute the interactions between these pathways: "The direction (import or export) and significance of these amino acid fluxes will be best determined e.g. with an inducible knockout mutant … to allow direct metabolic quantification of de novo synthesised amino acids". We nonetheless feel that such work goes beyond the scope of our present study.

Overexpression of the sequences in e coli would indicate function and could ensure that the enzymes were capable of operating with the correct direction (or both directions) for given substrates to product; however, the flux through any of these steps would not be known from such an activity analysis. Additionally, the flow through these steps is driven by concentration gradients (Hoppe et al., 1993; Stitt and ap Rees, 1979); thus, measurements of overexpression in e coli, and not isotope labeling of the diatom tissues would confirm a potential phosphoglyceromutase and enolase function but is unlikely to indicate anything further. I think the text on directionality (end of results) is appropriate and does not overstate the in vitro finding. As the authors point out, labeling would not inherently indicate the location of the production of PEP which is a focal point here, therefore even labeling analysis in diatoms may not be definitive unless the subcellular aspects could be resolved.

>>We thank the reviewer for their understanding of the problems associated with this question- the complexity of diatom metabolism renders the disentangling of individual metabolic fluxes challenging. In general, we have tried to emphasise gene expression data (e.g., expression of plastidial alanine transaminase and mitochondrial PGAM in qRT-PCRs shown in Fig. 4B) where we can at least associate the expression of a given gene with the metabolism of a particular organelle.

We have also clarified in the Discussion that our metabolic model (now Fig. 8) is based on the idea that triose phosphate, as generated by the Calvin cycle, is likely to be in significant stoichiometric excess to pyruvate in chloroplasts that are actively performing photosynthesis (lines 880-884): "Overall, our suggested roles for cpEnolase and cpPGAM1A are predominantly in favour of metabolic flux in the glycolytic direction … reflecting the innate metabolic activity of the Calvin cycle, which is likely to yield a high relative abundance of triose phosphate in the plastid under illuminated and photosynthetically active conditions". We have clarified (lines 888-892) that "These results are nonetheless inferential based … and would be more effectively validated via direct flux measurements" and also noted prior work from Moog et al suggesting the capacity of diatom plastids to readily import and/ or export PEP to clarify that the likely transport situation is more complex.

Are the claims fully supported? Do they move the field forward? The combination of phylogenetic analysis, subcellular localization prediction, and GFP localization provide sufficient evidence for me that the genes of interest are chloroplast-based.

The transcript levels of the genes in the field inferred from the Tara project and in the lab from knockout lines, are suggestive that they are actively expressed in conditions relevant to diatoms, though I was confused that changes to low temperature did not have an effect? Further, the lab diatom, presumably one

that was molecular biology friendly, is not native to polar regions. Thus, the claims related to this role in the wild are weakened accordingly and statements around figure 2 should be extremely cautious (see Fig 2 comment further down for clarifications). For reasons the authors indicate in rebuttal, performing competition assays would be challenging and in the discussion at 630 they indicate this remains to be determined. If Figure 2 cannot be better explained so that it shows diatoms that don't express these genes at other parts of the globe, then the characterization that these genes are important for sub-polar should be emphasized to be speculative.

>> We thank the author for their reasonable comments, and have addressed this question using three complementary approaches.

1) *We have reintegrated data from a previous iteration of our manuscript, where we used previously published transcriptome data (shown in a new Fig. S7) to explore the expression of* Phaeodactylum *copies of* cpEnolase *and* cpPGAM1A. *These data show rather clearly that neither gene is expressed in response to nutrient stress (including nitrate and phosphate starvation inferred to stimulate TAG accumulation and flux through acyl-coA synthesis pathways connected to glycolysis) but are transcriptionally upregulated in a light-sensitive manner, by long day (> 12h) illumination.*
2) *As no transcriptome data was to our knowledge previously available for* Phaeodactylum *grown at low temperature, we performed qRT-PCRs (shown in a new Fig. 2B) for empty vector cells grown under 19C LD, 19C CL and 8C CL conditions. These data show strong transcriptional induction (between 2- and 6-fold) of* cpEnolase *and* cpPGAM1A *in 19C CL compared to 19C LD conditions, and again (between 2 and 4-fold) between 8C CL and 19C CL conditions. Overall the relative expression of* cpEnolase *and* cpPGAM1A *are thus induced as much as 8-fold between 8C CL and 19C LD conditions, suggesting a physiologically significant role for this pathway in* Phaeodactylum *relating both to temperature and light. We describe both these points in a new Results section lines 262-363: "Plastidial localisation and expression dynamics of* Phaeodactylum *lower glycolysis enzymes". We have noted also in the Results (lines 532-533) that the conditions used for the subsequent physiology are environmentally relevant to* Phaeodactylum, *with the type strain used collected from the Irish Sea with annual sea temperature variation between 3 and 17C, and daylength variation 7 to 17 hours at the collection site latitude. Other* Phaeodactylum *ecotypes (e.g., Pt4) are known from the Baltic Sea, subject to even lower temperatures and seasonal ice coverage, and have further more been detect in the Arctic Ocean in Tara Oceans data (Sommeria-Klein et al., Science 2021) but have chosen not to discuss these data as they do not pertain to the Pt 1.86 type strain used in this study.*
3) *Finally, we have added a new Figure S12, exploring the distribution of plastidial glycolysis in diatom meta-genome assembled genomes (MAGs) where we can directly correlate occurrence to biogeography. While we do not interpret absence of evidence as evidence of absence, we note that diatom MAGs in which plastid glycolysis is not detectable uniquely do not have enriched distributions in high latitude stations; complementing previous data from cultured species (Fig. S1B) that only species containing clearly identifiable lower plastid glycolysis have been cultured from stations >60°N or >60°S. We additionally detect a possible independent recruitment of lower glycolysis to the chloroplasts of polar members of the chlorophyte genus* Micromonas, *which may be a more recent and therefore compelling example of an adaptive recruitment of this pathway. We detail these new findings in Results text lines 440-494: "Finally, we tested whether the occurrence of plastidial lower glycolysis may correlate to algal abundance at high latitudes. For this, we screened single-cell and meta-genome assembled genomes (sMAGs) from* Tara *Oceans … diatoms that possess complete lower plastidial glycolysis pathways shows positive correlations between mean mapped vertical coverage and absolute station latitude … No clear association between plastidial lower glycolysis and occupancy at high latitudes was observed for other algal groups, with the exception of chlorophytes".*

The included activity assessments establish function through production and purification from e coli, with the possible caveat that enzyme activities measured by spectrophotometer readouts of NADH could be insufficiently specific to the metabolism of interest. Nonetheless they are a supporting piece of evidence now provided.

>> As discussed in our response to Reviewer 1, we now use the same calculation method for the enzyme activity measurement as (Zhang et al 2020 Nature Communications), expressing enzyme activity by moles NADH consumption per enzyme mass per minute.

Figure 2: Its possible I am not following your relative comparison, but are there no significant amount of diatoms near the equator? (Suppl Fig S1 also) If there are why not show the distribution of all diatoms and their expression within this picture, otherwise there is no reason to know if there are other diatoms at other places and whether they use these genes in their habitats? Its not compelling unless the diatoms expressing these genes are plotted relative to others not expressing these genes in warm environments. You indicate they are relative, but I don't see all diatoms are being plotted to show others for example near the equator that are not expressing the genes? Fig S7 is not helping my understanding of this. What you want to show is that the expression pattern in diatoms across the globe and support that expression is mostly in the sub-polar regions, but I do not understand what size fraction means and therefore I cannot interpret even the yellow vs orange and size of the bubbles in pictures like S7iii that seem to show some level of enolase near the equator and non-polar regions. What do size fractions in um have to do with expression of a gene?

>> We apologise for the over-simplified view of our data, which rather reflects how Tara *Oceans distributions are typically presented for specialist oceanographer and marine biology readers.*

In effect, diatoms are more abundant in high latitude than temperate and tropical stations, and this may contribute to the greater occurrence of the meta-transcript sequences of cpEnolase *and* cpPGAM *genes at these stations. Nonetheless, we can quantify a greater relative expression of these genes in high latitude regions through several different strategies:*

- *First, as shown in Fig. 3, while we see a slight increase in diatom DNA (metaG) sequence abundance for these genes at high latitudes, this is dwarfed by the dramatic increase in RNA (metaT) sequence abundance. We prefer to plot these separately so that the reader can visualise both the metaT and metaG abundances, and to avoid scaling errors that could be introduced by dividing metaT over metaG in stations where zero metaG abundances are detected. Nonetheless, to make this figure easier to follow, we have directly positioned the metaT plot for each gene above its corresponding metaG plot to allow easy visual comparison of each distribution. We have also added plots for DCM (deep chlorophyll maximum) depths for each gene, where the latitudinal induction of metaT data only is even more visible.*
- *Alongside this, we have performed extensive correlation tests on different normalisations of our data, to assess whether there is a significant association between* cpEnolase *and* cpPGAM1 *expression and latitude. Alongside comparing metaT to metaG as alluded to in Fig. 3, the key comparison that we perform is by dividing* cpEnolase *or* cpPGAM1 *metaT by all diatom metaT transcripts, i.e., to identify what % of the diatom transcriptome is occupied by each gene. We now show these normalisations in a new fig.S10 that show significant positive correlations between diatom chloroplast relative* cpEnolase *and* cpPGAM1A *metaT and latitude, and describe explicitly what these plots show in a new Results text lines 387-415: "To confirm that this was due to a greater expression of* cpPGAM *and* cpEnolase *genes, as opposed to being purely driven by the greater relative abundance of diatoms in high latitude* Tara *Oceans stations, we performed multiple normalization tests (Fig. S10; Supplemental Dataset S3, sheet 10). First, metaT abundances calculated for each gene in the 0.8-2000 μm size fraction were divided by the total relative abundance of all diatom metaT sequences, providing the total proportion of each diatom meta-transcriptome occupied by* cpEnolase *and* cpPGAM*. … Alongside this, the metaT abundances obtained for diatom* cpEnolase *and* cpPGAM *genes were compared (via log normalisation, to allow the inclusion of zero values) to the relative abundances calculated for the meta-genomic (metaG) sequences of the same genes … ranked (Spearman) correlation values were also calculated for normalised chloroplast glycolysis metaT expression levels. Significant positive correlations with latitude were detected…"*
- *Alongside this, and as alluded to by Reviewers 3 and 4, we perform similar calculations for individual depths and cell sizes (size fractions) in* Tara *Oceans data, as shown in Fig. S9 (previous Figure S7). The size fractions do not relate themselves to gene expression but, as described above, allow us to determine whether the same signal is detectable consistently in diatoms from the*

micro- to macro-scale, and in diatoms living in the surface and in lowly lit but nutrient- and chlorophyll-rich deep waters. We explain this in the Results text lines 380-382: "We observed these trends concordantly in both surface and deep chlorophyll maximum (DCM) samples from … individual size fractions … suggesting broad reproducibiilty across diatoms independent of cell size and depth" and again in lines 425-426 "with significant positive correlations to latitude detected in multiple individual size fractions and depths", and finally in the Fig. S9 legend: "These data, which allow us to identify whether specific trends are observed in different kinds of diatom cells, ranging from the nano to micro-metre scale, support global trends observed across all size fractions and surface layers

- *Finally, we now look into meta-genome assembled genomes from Tara Oceans (Fig. S12; Delmont et al. 2022), where we can plot the effective species ranges of diatoms that can be detected in meta-genomic data, and correlate these to presence or absence of detectable plastidial glycolysis. As discussed, diatoms that possess detectable plastidial glycolysis enzymes show greater mapped genome sequence incorporation in high latitude stations, while those that lack them do not. This is particularly interesting insofar as other algal groups in which lower plastidial glycolysis is detectable (haptophytes, pelagophytes, chrysophytes) largely do not show the same latitudinal association amongst their MAGs, suggesting that the association of lower plastidial glycolysis with greater occupancy of high latitude stations is principally a diatom-associated feature. We nonetheless describe a probable recruitment of lower half glycolysis to the chloroplasts of high latitude ecotypes of the distantly related green alga Micromonas, as inferred from MAG data, suggesting that this pathway may have been independently associated to high latitude adapted algae in other branches of the tree of life.*

Figure 3: not particularly compelling that light or temperature matters? Further at 8C, there is no difference - a cold condition... yet the KO line is a transformable line...and not a sub-polar line. I think at MOST you can infer from figure 2 a role in sub-polar conditions in the discussion and word carefully in results, but I remain skeptical of that role similar to prior reviews.

>> We feel that our data rather strongly position light as having a strong physiological impact on our glycolysis knockout lines, with a difference between 10 and 30% reduction in growth rate under light: dark cycling versus continuous light conditions. We have supported this with evidence from published sequence data (Fig. S7A) that both cpEnolase and cpPGAM1A are overexpressed under long-day conditions and qRT-PCR of wild-type cells (Fig. S7B) showing transcitptional upregulation of both cpEnolase and cpPGAM1A under continuous light, compared to light: dark cycling.

We agree that there is no clear difference in growth rate between our knockout and control lines at 8C- which is perhaps a reflection of the overall lower growth rate observed across all lines- but the high levels of expression of cpEnolase *and* cpPGAM1A *under these conditions (Fig. 2B) and the complex gene expression, metabolite and lipid accumulation phenotypes observed in knockout lines (Figs. 5B, 6 and 7) point to significant probable flux through this pathway at low temperatures. We respectfully do not agree that an absence of a growth difference indicates an absence of any phenotype- it is quite possible that plastid glycolysis may play important roles in diatom physiology whose absence may even be deleterious under these conditions in the wild, but with laboratory growth rates buffered by the light field, nutrient regime and duration of culture in exponential phase. We explain this in the Discussion lines 867-868: "We note that the relative expression of* cpEnolase *and* cpPGAM1A *are even greater at 8C than 19C under continuous light conditions, and it is possible that flux occurs through plastid glycolysis at low temperature despite the absence of a clear growth phenotype in knockout versus control lines" and 1023-1024: "It also remains to be determined how these functions impact growth kinetics and viability of diatoms in the wild, given the limited differences in growth rate observed in the lab between knockout and control lines"*

Figure 7B: consider just combining with 7A as bar plot activities - you cannot infer flux, as the triose phosphate interconversion is driven by concentration gradients which are unknown and would vary with condition. Plot the activities. Further, I think the point is that they have activity consistent with putative gene function of PGAM/ENO, not really that you can assess their reversibility in a biological context specific to a diatom in a particular environment, through an activity assay.

Its unfortunate you were unable to measure the substrates or products of the genes for the knockout relative to the WT, this could have been helpful, if they were being significantly used, and further could have supported your assessments for the role of these genes in cool conditions. Of course, metabolite levels may not be indicative of metabolism generally; but in a KO the immediate substrate and product are often affected. Fortunately, at least lower levels of pyruvate were indicated in the KO.

>> As discussed above in our response to Reviewer 3, we feel that the most important component of the enzymatic activity was to determine reversibility of the enzymes as opposed to inferring in vivo biochemical functions, and that the potential directions and significance of each enzyme can be inferred through multiple phenotypes observed in our knockout lines. We summarise this evidence in the Results and Discussion:

- *Lines 881-885: we note, as pointed out by the reviewer, that the knockout lines substantially underaccumulate pyruvate, on the acceptor side of the glycolytic reaction of cpEnolase; and further that the* cpEnolase *mutants only overaccumulate 3-phospho-glycerate, which may be synthesised from triose phosphate. We consider that these are both strong pointers to impeded flux in the glycolytic direction.*
- *Lines 896-913: we further identify phenotypes that relate to enzymatic activities associated with one organelle only, e.g., a downregulation of shikimate biosynthesis and upregulation of mitochondrial glycolysis, which point, respectively, to an underaccumulation of plastidial PEP and overaccumulation of exported triose phosphate, thus probable glycolytic flux through plastidial glycolysis under 19C LD conditions.*
- *Lines 913-933: we similarly note lipidomic features, e.g., an underaccumulation of TAGs under 19C CL, which in our opinion strongly points to impeded production of plastidial glycolysis in knockout lines*
- *Our suggestion of increased flux through plastidial glycolysis in 19C CL conditions relates not only to the increased growth defect in knockout lines, and the much more dramatic reorganisations of cellular transcription observed in lines, but also to the greater expression of* cpEnolase *and* cpPGAM1A *under these conditions. We discuss this in lines 913-990, including : "These are notably concordant with greater expression of* cpEnolase *and* cpPGAM1A *in wild-type cells, and an enhanced growth defect in knockout lines, together suggesting greater potential flux through this pathway than under 19C LD"*
- *Finally, our suggestion of a more complex flux at 8C not only relates to growth rate but to the broader metabolic phenotypes observed, as noted in lines 996-9997: "Knockout lines under 8C conditions, however, have additional phenotypes not observed at 19C". These include "an overaccumulation of TAGs and SGDQs over glucosyl-lipids, and the over-accumulation of aspartate and phenylalanine in* cpEnolase *knockouts only". We propose that these phenotypes, can be most logically explained by an increase in acyl-coA synthesis from plastidial pyruvate; and from metabolic pathways accessible from plastidial PEP only, respectively. We feel that this is a strong argument for a gluconeogenic activity under these conditions, although note for balance in lines 983-984: "it remains to be determined to what extent these enzymes function bidirectionally in vivo".*

Concerning Fig. 8 (was Fig. 7), we agree that a single panel figure would be more informative. Nonetheless, we feel it is important that there is some degree of visual guide for what we propose in terms of the activity of each enzyme, particularly for readers who are specialised in evolutionary biology or ecology, but may otherwise not be able to join the dots between the rather large amount of information presented in the Discussion. We agree that we cannot exclude a metabolic activity in either direction in any condition, so we have made the reaction figures for each condition bidirectional, merely showing that at 19C we see stronger evidence for activity in the glycolytic than gluconeogenic direction, at 8C we see some unique features implying possible gluconeogenic activities, and overall we see stronger phenotypes in CL than LD conditions. We have also explicitly noted in the Figure 8 legend that the figure shows "Proposed activities" and "potential inferred roles" of plastid glycolysis.

Minor:

A. I would alter the title to be something about the triose conversions of glycolysis to pyruvate in chloroplasts of diatoms?

>> We have modified the title to "Complementary environmental data analysis and functional characterization of a plastid diatom lower glycolytic-gluconeogenesis pathway" to confirm that we are not discussing upper half glycolysis. We feel that explicitly mentioning pyruvate is inappropriate as the enzymatic activities we have studied specifically act between 3-phosphoglycerate and PEP

B. Abstract 49: should state specifically 'for diatoms', we don't know what other species might have green chloroplasts with ENO/PGAM and whether they inherited from mitochondria, right?

>> In effect this evolutionary history is explored in Figs. 1-S4 for pelagophytes, chrysophytes, haptophytes etc, but we have added "diatoms" for ease of reader interpretation

C. Understanding of gluconeogenesis is not correct in line 72, gluconeogenesis is not for the production of starch rather sugars, consider phrasing carefully.

>> We have clarified "gluconeogenesis of glucose-6-phosphate"

D. Figure 1D: yellow is green and red, but would be better to see each color and then merge at least for the example shown, rather than put in S5? I think this would make it easier for the reader.

>> We agree and have moved the GFP images, under separate channels, to Fig. 2 panel A to allow us to show it to full scale in one page.

E. Figure 3: specify the control is empty vector and not just WT.

>> Modified.

Dear Dr. Richard Dorrell:

We have received reviews of your manuscript entitled "Complementary environmental analysis and functional characterization of a plastid diatom lower glycolytic-gluconeogenesis pathway." On the basis of the advice received, the board of reviewing editors would like to accept your manuscript for publication in The Plant Cell. This acceptance is contingent on revision based on the comments of our reviewers. In particular, please consider the minor suggestions from reviewer 5.

Please highlight all changes and include a detailed annotation of changes of the text, with line numbers, and noting your responses to the comments.

To submit your revised manuscript, click:

Link Not Available

If you have any questions about the revision submission procedures, please contact the Editorial Office Staff (tpc-submissions@aspb.org). If you cannot return the revised manuscript within 30 days, please let us know. Otherwise, we will assume that you have elected not to revise the manuscript and withdraw it.

Thank you very much for the privilege of reviewing this work. I look forward to receiving the next version.

Sincerely,

The Plant Cell Board of Editors

Reviewer #3 (Comments for the Author):

The authors in my opinion now have addressed successfully all critical aspects.

Reviewer #5 (Comments for the Author):

Thank you to the authors for the revisions and detailed explanations. The reworked description addresses my largest concerns and provides some clarity on aspects that were confusing. The authors acknowledge that diatom presence globally is concentrated in high latitudes and report a relative comparison of transcripts, in essence to normalize so the cpPGAM or cpENOLASE can be compared to other expressions. This is likely the best that can be done in this circumstance; and comparison to southern temperate latitudes further supports findings. I have one remaining small suggestion for the supplements and a few typographical corrections.

188: As most crops are angiosperms, it would be informative to know what species of angiosperms contain either PGAM or enolase or both in the chloroplast, but I could not easily deduce that from S1 or the S1 data, unless I missed this. Can you provide a list of all of the individual species surveyed as part of the supplement, instead of grouping by class? I do appreciate that the role in diatoms seems to be a deviation from other photosynthetic systems more generally, nonetheless the extensive characterization performed does not seem to be fully accessible in the supplements as provided, unless I am missing something.

145: 'in' organelles
458: typo "fiuve"
471: typo "lartitude"

Please also note the following:

-The Plant Cell now requires authors to complete and submit an author revisions checklist upon submission of a revised manuscript. The aim of the checklist is to aid authors in preparing a high-quality manuscript, facilitate the review and

assessment of revised manuscripts, and help to ensure that journal standards are maintained across the board. If your manuscript is accepted, the completed checklist will be published as supplemental material attached to the article online. Please download a copy of the checklist (pdf fillable form) at this link, for submission with your revised manuscript: https://tpc.msubmit.net/html/Author_Revisions_Checklist.pdf.

-Supplemental materials should be restricted to large datasets and tables, presentation of replicates, and validation of reagents, methods, or genotypes. Any data that are used to support the major claims must be in the main manuscript. Supplemental figure legends must indicate what figure in the main manuscript is supported by the supplemental data presented. Please justify how each of the supplemental figures meet the criteria.

-Sampling methods and the nature of "biological replicates" should be described precisely (i.e. different plants, parts of plants, pooled tissue, independent pools of tissue, sampled at different times, etc.), along with a clear description of and rationale for any statistical analyses conducted. The reader should know exactly what was sampled; what forms the basis of the calculation of any means and statistical parameters reported. This is also necessary to ensure that proper statistical analysis was conducted.

Dear editors,

We thank you and the reviewers for the time and careful comments applied to our manuscript. As per our previous resubmission, we provide a detailed response to the supplied comments below in italic text with a double caret (“>>”) indent. We have confirmed that all of the new data associated with this revision are appropriately documented, and resubmit our previous copy of the Author Revision Checklist without modification from the primary resubmission.

Dear Dr. Richard Dorrell:

We have received reviews of your manuscript entitled "Complementary environmental analysis and functional characterization of a plastid diatom lower glycolytic-gluconeogenesis pathway." On the basis of the advice received, the board of reviewing editors would like to accept your manuscript for publication in The Plant Cell. This acceptance is contingent on revision based on the comments of our reviewers. In particular, please consider the minor suggestions from reviewer 5.

Reviewer #5 (Comments for the Author):

Thank you to the authors for the revisions and detailed explanations. The reworked description addresses my largest concerns and provides some clarity on aspects that were confusing. The authors acknowledge that diatom presence globally is concentrated in high latitudes and report a relative comparison of transcripts, in essence to normalize so the cpPGAM or cpENOLASE can be compared to other expressions. This is likely the best that can be done in this circumstance; and comparison to southern temperate latitudes further supports findings. I have one remaining small suggestion for the supplements and a few typographical corrections.

188: As most crops are angiosperms, it would be informative to know what species of angiosperms contain either PGAM or enolase or both in the chloroplast, but I could not easily deduce that from S1 or the S1 data, unless I missed this. Can you provide a list of all of the individual species surveyed as part of the supplement, instead of grouping by class? I do appreciate that the role in diatoms seems to be a deviation from other photosynthetic systems more generally, nonetheless the extensive characterization performed does not seem to be fully accessible in the supplements as provided, unless I am missing something.

>>We have added a species list with tabulated numbers of Enolase and PGAM enzymes, including inferred plastid-targeted copies of each, in Table S1, sheet 2, and an interactive tree format of these distributions (OneKp data only) in Table S1, sheet 3. We have clarified this in lines 180-187: “An overview of the distributions of these proteins in different taxonomic groups of plants and algae is provided in Table S1, sheet 1; a complete list of species with all inferred plastid and non-plastid targeted copies of these proteins is provided in Table S1, sheet 2; and the complete OneKp ASTRAL topology labelled with the presence of detected plastid-targeted glycolysis proteins is provided in Table S1, sheet 3.”

We have also noted (lines 196-201) concerning the distribution of probable angiosperm plastid-targeted lower-half glycolysis-gluconeogenesis that: “Alongside the previously described Arabidopsis thaliana plastid lower-glycolytic pathway, these species were found in a wide range of different plant families, with the greatest numbers in Lamiales (7 species with both plastid-targeted enolase and PGAM), Asterales (5 species) and Malphigiales (4 species), potentially indicating recurrent origins of this pathway in higher plants (Table S1, sheets 2 and 3).”. We regret that our source data for this analysis, OneKp, contains very few crop species, and we are unable to detect any economically important species in our data that possess both cpEnolase and cpPGAM enyzymes.

145: 'in' organelles
458: typo "fiuve"
471: typo "lartitude"

>>*We thank the reviewer for the careful reading of our manuscript, and have corrected all three noted errors.*

Dear Dr. Richard Dorrell:

We are pleased to inform you that your paper entitled "Complementary environmental analysis and functional characterization of a plastid diatom lower glycolytic-gluconeogenesis pathway" has been accepted for publication in The Plant Cell, pending a final editorial review by a science editor. At this stage, your manuscript will be evaluated by a science editor with respect to its presentation of scientific content, compliance with journal policies, and presentation for a broad readership. The Plant Cell has contracted with Plant Editors (planteditors.com) to provide this service to our authors, and you will soon receive additional information on this process.

Please note that each author needs to link their ORCID identifier to their account in the system before your manuscript can be published. If any authors do not have an ORCID linked to their account, they will receive a message with a link to complete this task. Please ensure that ALL of your coauthors have completed this task as soon as possible.

ASPB offers an OPEN option that allows authors to have their online articles available for free to all users immediately upon publication. For more information about the ASPB OPEN option, refer to the Final Submission Checklist Form.

The Plant Cell and The Arabidopsis Information Resource (TAIR) are collaborating to collect functional annotation data about Arabidopsis genes from authors. This includes information about the gene's molecular function (e.g., kinase activity, ATP synthetase activity), the biological process/es it is involved in (e.g., endosperm development, threonine biosynthesis), its subcellular location (e.g., nucleus, ER), anatomical or developmental expression pattern (e.g., leaf, ovule, flower stage 10, seedling stage), or its partner in a protein-protein interaction (e.g., AT1G01010 interacts with AT1G01020).

If your paper contains results falling into one or more of these categories for Arabidopsis genes, we request that you now submit these data for inclusion in TAIR by filling in the form provided at the following URL: https://www.arabidopsis.org/doc/submit/functional_annotation/123. If you need further clarification on what types of data can be submitted please contact curator@arabidopsis.org.

Finally, we encourage your submission of artwork for the journal cover. Monthly issues will have an online cover image and selected covers will be used for posters, other promotional items, and "wallpaper" for mobile devices. For more information, refer to Cover Submission in the Instructions for Authors https://tpc.msubmit.net/cgi-bin/main.plex?form_type=display_auth_instructions.

We look forward to seeing your paper published.

Sincerely,

The Plant Cell Board of Editors

=====
IMPORTANT REMINDER: PEER REVIEW REPORTS
=====

If you opted to publish a peer review report along with your article during the original submission process, it will be prepared by the editorial staff and publicly posted with your manuscript, inside the zip file that contains any other supplemental material. As a reminder, the peer review report is a public record of all comments from editors and reviewers, as well as your prior responses, as you received them in the decision letters for each draft of your manuscript. If you agreed to publish this report and have changed your mind, or are not sure if you selected this option, please contact the editorial office as soon as possible before signing the license agreement from our publisher.

----FOR ASPB OFFICE USE ONLY (DO NOT EDIT)----
MSID: 39058
Corresponding Author: Dr. Dorrell
